# Misplaced Central Venous Catheter Leading to a Chemothorax: Case Report

**DOI:** 10.5811/cpcem.62200

**Published:** 2026-08-05

**Authors:** Hannah E. Miller, Skyler Lentz

**Affiliations:** * University of Vermont Health, Department of Emergency Medicine, Burlington, Vermont; † University of Vermont Health, Department of Emergency Medicine, Division of Resuscitation Science, Burlington, Vermont; ‡ The Robert Larner, M.D. College of Medicine at the University of Vermont, Burlington, Vermont

**Keywords:** chemothorax, malposition of central venous catheter, pleural effusion, case report

## Abstract

**Introduction:**

The placement of central venous catheters (CVC) is a common procedure for the administration of chemotherapy. Adverse events include malposition or displacement; there are limited reports of misplaced implanted CVCs.

**Case Report:**

A 59-year-old female with a history of recently diagnosed metastatic small cell lung cancer, hypertension, former tobacco use of over 60 pack-years, and chronic obstructive pulmonary disease without chronic hypoxemic respiratory failure presented to the emergency department following the first outpatient infusion of chemotherapy. She developed acute onset dyspnea, moderate respiratory distress, and hypoxemia. She was found to have a malpositioned subclavian implanted (ie, “port”) CVC that was placed under fluoroscopy the week prior with placement confirmed by chest radiograph. The evaluation revealed a large right pleural effusion secondary to unintentional intrapleural infusion of chemotherapy. The patient was transferred to a tertiary-care center with successful clinical improvement after drainage of the effusion via tube thoracostomy.

**Conclusion:**

We present an uncommon diagnosis of a chemothorax from a malpositioned central venous catheter in the thorax, despite a radiograph suggesting correct placement, and infusion of chemotherapy causing a common complaint of shortness of breath. This case highlights the limitations of radiographic confirmation of CVCs and a resultant complication from malpositioning.

## INTRODUCTION

It is commonplace to encounter patients who have established placement of central access with an implanted (ie, “port”) central venous catheter (CVC) to facilitate regular infusions of chemotherapy. After placement of a CVC, it is routine practice to confirm catheter location with an anteroposterior plain radiograph of the chest, which can also detect immediate complications such as pneumothorax, hemothorax, or effusions. There are few recorded incidences of misplacement and/or displacement of port CVCs.^1^–^5^ Less common examples include placement in an extracava space, which includes the epidural space, pericardium, pleural space, mediastinum, or thoracic duct.^6^ Misplacement of these catheters can lead to serious adverse events because the medications administered through these venous access. devices are potent and potentially harmful and caustic to peripheral vessels or body cavities.

## CASE REPORT

**Image 1:**
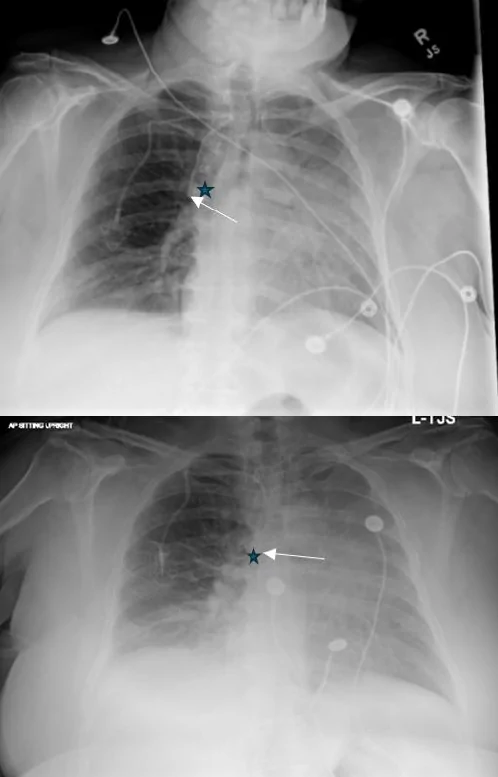
Top: Anteroposterior (AP) view chest radiograph immediately after central venous catheter placement, demonstrating radiologic confirmation of appropriate placement with the tip overlying the expected location of the lower superior vena cava. Bottom: AP view chest radiograph upon presentation to the emergency department. Arrow is pointing to the tip of the central venous catheter. Star indicates the location of the superior vena cava, which is located to the right side of the mediastinum above and below the level of the carina.

A 59-year-old female with a history of recently diagnosed metastatic small cell lung cancer with malignant obstruction of the left upper lobe, hypertension, former tobacco use of over 60-pack-years, and chronic obstructive pulmonary disease (COPD) without chronic hypoxemic respiratory failure presented to the emergency department (ED) following the first outpatient infusion of chemotherapy. She developed acute-onset dyspnea, moderate respiratory distress, and hypoxemia. The patient had a right subclavian approach implanted central venous catheter placed under fluoroscopy six days prior with radiographic placement confirmation (Image 1).

*CPC-EM Capsule*

What do we already know about this clinical entity?

*A chemothorax is the infusion of chemotherapy into the pleural space.*

What makes this presentation of disease reportable?

*A misplaced central venous catheter (CVC) can lead to the uncommon diagnosis of chemothorax.*

What is the major learning point?

*There are limitations to routine forms of radiographic confirmation for central venous catheter placement.*

How might this improve emergency medicine practice?

*A misplaced CVC leading to a chemothorax should be considered in the setting of a new onset pleural effusion after an infusion through a recently placed CVC.*

In the ED, the initial vital signs were as follows: blood pressure, 118/71 mm Hg; pulse, 71 beats per minute; oral temperature, 36.7 °C (98 °F); respiratory rate, 24 breaths per minute; and pulse oximetry, 96% on 2 L of oxygen delivered by nasal cannula, with a saturation of 80% on room air before supplemental oxygen. The physical examination demonstrated that the patient was in moderate respiratory distress with wheezes in all lung fields upon expiration, pursed lip breathing, and increased accessory muscle use. The patient was initially in sinus rhythm but developed atrial fibrillation with rapid ventricular response. Nebulized albuterol and ipratropium, corticosteroids, and antibiotics were administered for possible COPD exacerbation, furosemide for possible fluid overload, amiodarone 150 mg bolus and infusion at 1 mg per minute and 2 g magnesium sulfate for atrial fibrillation with rapid ventricular response. Of note, all medications were given through the port CVC.

**Image 2:**
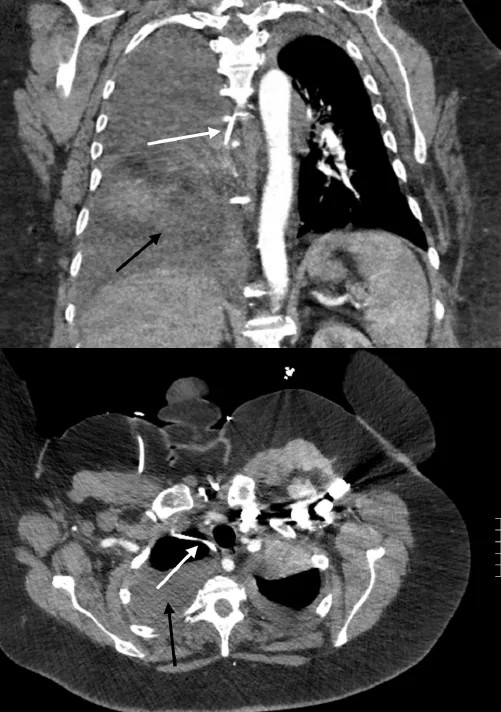
Sagittal (top) and axial (bottom) views from a contrast-enhanced chest computed tomography chest demonstrating a large right pleural effusion (black arrows) adjacent to a central venous catheter (white arrows) of uncertain location.

Laboratory studies were notable for an elevated d-dimer of 671 ng/mL (reference range, < 230 ng/mL); venous blood gas with pH 7.27 (7.31–7.41) and partial pressure of carbon dioxide 56 mm Hg (41–51 mm Hg); an undetectable troponin; and N-terminal pro B-type natriuretic peptide 920 pg/mL (< 221 pg/mL). Chest radiograph revealed no pneumothorax, persistent opacification of the left upper lobe, and a new patchy opacification of the right lower lobe associated with blunting of the right costophrenic angle (Image 1). Point-of-care ultrasound (POCUS) demonstrated no evidence of pulmonary edema, normal left ventricular ejection fraction, no pericardial effusion, small pleural effusion at the left lung base, and a large pleural effusion at the right lung base. Computed topography pulmonary angiogram revealed a large right pleural effusion with malpositioning of the right port CVC with inability to determine whether it was intravascular. There was interval development of incomplete collapse and consolidation of the right lower lobe, likely due to compression from the large right pleural effusion (Image 2).

Medication administration was switched to peripheral intravenous cannula. The transfer process to a tertiary-care center for higher level of care was initiated. The patient’s respiratory status ultimately improved with decompression of the pleural effusion via placement of a pigtail pleural catheter in the intensive care unit. The pleural fluid analysis showed a pH of 7.45, total protein concentration < 2.0 g/dL, and an immeasurably low lactate dehydrogenase, consistent with a transudative effusion. The pathology of the fluid demonstrated reactive mesothelial cells. The patient did initially require noninvasive positive pressure ventilation; although, by the end of her hospital stay, the patient no longer required oxygen supplementation. The patient was discharged with prescriptions for anticoagulation and amiodarone to manage her atrial fibrillation. The subclavian CVC was removed by interventional radiology after a venogram confirmed malposition. The venogram demonstrated that the port catheter did not pass through the right subclavian vein. A peripherally inserted central catheter was placed for future chemotherapy infusions.

## DISCUSSION

This case contributes to emergency medicine and critical care fundamental knowledge by highlighting the limitations of radiographic confirmation when evaluating CVC placement. It is routine practice to obtain an anteroposterior chest radiograph to assess for correct placement and for presence of an immediate complication after placing an internal jugular vein or subclavian vein CVC.^7^ However, there is no general consensus on what a universally accepted CVC tip position is.^7^ There are no established landmarks that are officially used for describing appropriate position; although, the carina is a good estimation of where the distal SVC lies just above the right atrium.^8^ Further, anteroposterior radiographs are limited by their two-dimensional anatomic visualization, rendering a limited assessment for extravascular malpositioning if it occurs in the expected location of the SVC.^9^ Of note, studies have shown the utility of real-time thoracic ultrasound in confirming placement of CVC by visualizing the guidewire in the right heart chambers.^10^ This method of dynamic confirmation may be more definitive for the confirmation of CVC positioning by clinicians facile with point-of-care echocardiography.

Chemothorax is a rare complication in which chemotherapy is accidentally infused into the pleural space due to catheter malposition.^11^ The chemotherapeutic agent is potentially destructive or inflammatory to the delicate lung tissue, resulting in combined systemic and local-regional toxic effects.^12^,^13^ The treatment is prompt pleural drainage via thoracentesis or pleural catheter to limit the risk of toxic effects and to relieve the symptoms of dyspnea and hypoxemia caused by collapse of the lung, or atelectasis, resulting in a ventilation-perfusion mismatch. The proper handling of chemotherapeutic agents should be considered when the fluid is drained from the pleural space. In this patient case, it is unclear how much damage, if any, was done to the lung parenchymal tissue by the chemotherapeutic agents. The transudative nature of the pleural fluid suggests against severe inflammation. Prior cases of chemothorax described in case reports presented similarly with development of dyspnea during or shortly after chemotherapy infusion and with initial fluid studies demonstrating transudative effusion.^2^,^11^,^14^ Outcomes were favorable after removal of the foreign substance by pleural drainage. Further case comparisons are limited given the scarcity of reported cases of chemothorax.

In this case, we set out to treat and rule out the common reasons for acute onset of shortness of breath. In doing so, the true diagnosis, a chemothorax, and the reason for it, a misplaced CVC, were discovered incidentally. This case describes the importance of considering the adverse event of misplacement of a port CVC, even when placement is considered confirmed by chest radiograph, in the setting of new-onset shortness of breath during, or shortly after, initiation of chemotherapy infusion.

## CONCLUSION

This case highlights the limitations of radiographic confirmation of central venous catheters. We present an uncommon diagnosis of a chemothorax from a malpositioned CVC in the thorax, despite prior confirmed placement, and an infusion of chemotherapy causing shortness of breath. A misplaced CVC leading to a chemothorax should be considered in the setting of a new-onset pleural effusion seen on chest radiograph or point-of-care ultrasound after an infusion through a recently placed CVC. The management includes discontinuing use of the central catheter, drainage of the pleural effusion, and expert removal of the catheter.
